# A preliminary assessment of low level arsenic exposure and diabetes mellitus in Cyprus

**DOI:** 10.1186/1471-2458-12-334

**Published:** 2012-07-04

**Authors:** Konstantinos C Makris, Costas A Christophi, Martha Paisi, Adrienne S Ettinger

**Affiliations:** 1 Cyprus International Institute for Environmental and Public Health in association with Harvard School of Public Health, Cyprus University of Technology, Irenes 95, Limassol, 3041, Cyprus; 2 Center for Perinatal, Pediatric & Environmental Epidemiology, Division of Chronic Disease Epidemiology, Schools of Medicine and Public Health, Yale University, New Haven, CT, USA

**Keywords:** Arsenic, Diabetes, Exposure, Drinking water, Environmental health, Endocrine

## Abstract

**Background:**

A preliminary study was undertaken in a community of Cyprus where low-level arsenic (As) concentrations were recently detected in the groundwater that was chronically used to satisfy potable needs of the community. The main objective of the study was to assess the degree of association between orally-ingested As and self-reported type-2 diabetes mellitus (DM) in 317 adult (≥18 years old) volunteers.

**Methods:**

Cumulative lifetime As exposure (CLAEX) (mg As) was calculated using the median As concentrations in water, individual reported daily water consumption rates, and lifetime exposure duration. Logistic regression models were used to model the probability of self-reported DM and calculate odds ratios (OR) in univariate and multivariate models.

**Results:**

Significantly higher (p *<* 0.02) CLAEX values were reported for the diabetics (median = 999 mg As) versus non-diabetics (median = 573 mg As), suggesting that As exposure could perhaps be related to the prevalence of DM in the study area, which was 6.6%. The OR for DM, comparing participants in the 80^th^ versus the 20^th^ percentiles of low-level As CLAEX index values, was 5.0 (1.03, 24.17), but after adjusting for age, sex, smoking, education, and fish consumption, the As exposure effect on DM was not significant.

**Conclusions:**

Further research is needed to improve As exposure assessment for the entire Cypriot population while assessing the exact relationship between low-level As exposure and DM.

## Background

Arsenic (As) is a known Group A human carcinogen that has been associated with carcinogenic and non-carcinogenic effects in numerous epidemiologic studies around the world [1-8]. Arsenic carcinogenicity to living organisms, including humans, has been well established [3]. During the last decade, a considerable shift has been directed towards the study of non-carcinogenic effects of As, such as dermal effects (hyperpigmentation and keratosis), adverse pregnancy outcomes, hypertension, peripheral vascular disease, cardiovascular diseases, respiratory diseases (asthma), immune response, and type-2 diabetes mellitus (DM) [1-8]. Even though the dose response relationships for these various non-carcinogenic end points seem to be well-resolved for relatively high As exposures, i.e., > 100 μg L^-1^, the shape and magnitude of the dose–response curve for low-level As exposures (>10 – 100 μg L^-1^) is unclear. This is also the case for DM, where low level As effects on the prevalence of DM have yet to be fully elucidated.

Human physiologic abnormalities associated with insulin resistance or the inability to produce enough insulin, an essential hormone used to regulate blood glucose levels and maintain cell metabolism, are reaching epidemic dimensions, worldwide. Type-2 diabetes mellitus is predicted to affect a total of 366 million people by 2030, rising from a worldwide estimate of 171 million people in 2000 [9]. Known risk factors for DM include: obesity, older age, physical inactivity, family history, and genetic polymorphisms [10]. Environmental toxicants, such as As, have been implicated in the etiology of DM in epidemiologic and animal studies [11,12]. Relatively high exposure scenarios (> 100 μg L^-1^) have been studied in epidemiologic studies [5,13,14]. whereas only a few have focused on low-level As exposures (< 100 μg L^-1^) [15,16].

The scarcity of available data on the relationship between low level As exposure and DM led us to investigate this further, by selecting a specific area of Cyprus that had been chronically exposed to As in groundwater used for potable purposes. During the last two decades, DM has been a major determinant of the overall burden of disease in Cyprus [17]. Recent data from the only available epidemiologic study in Cyprus showed that 6.5% of the adult population (between 20–80 years old) had DM, while there was another estimated 3.8% of the adult population who remained undiagnosed [17].

The main objective of this study was to assess the degree of association between orally-ingested As and self-reported DM in a specific area of Cyprus. A detailed questionnaire was completed by 414 residents of the As-affected community in Cyprus, and data on demographic characteristics, water use habits, and medical history were also collected.

## Methods

### Study location and characteristics

The small community of Mammari (~ 1600 residents) (CENSUS data) in the Nicosia prefecture of Cyprus was chosen for this study, because of recently documented groundwater pollution with As that was used to satisfy the area’s potable needs. During the summer of 2009, regulatory agencies of the Cypriot government detected As concentrations in samples of the groundwater and home tap water in the Mammari area, exceeding the maximum contaminant level value of As in drinking-water (10 μg L^-1^). Uncertainties associated with the duration of exposure, magnitude of As intake, and the possible health effects largely prevailed. A wide range of As concentrations in the region’s groundwater has been observed (< 10 – 70 μg L^-1^ As), and a value of ~40 μg L^-1^ was chosen for the calculation of an individual’s daily As intake.

This study was undertaken in parallel with that of a national scientific committee on As health effects organized by the Cypriot Ministry of Health. Our study’s collection of questionnaires and data protection was accompanied by signed informed consent from each participant and all participants were able to ask questions about the study. The signed informed consent mentioned that participants agreed to disclose personal data for the study analyses, while full review was not required, since no biological tissue sampling was done and no external funding was involved. Our study approach followed the same guidelines of the bioethics protocol used by Harvard School of Public Health to conduct the Kuwait Pilot Medical Monitoring Study (D. Christiani, Harvard School of Public Health, personal communication). The As-affected area’s local administrative council (Mammari, Cyprus) approved the study and it was held responsible for distributing questionnaires to all of their residents. The questionnaire was based on the original questionnaire used in the Kuwait Pilot Medical Monitoring Study and it was modified to account for the specific lifestyle habits of Cyprus. The study questionnaire focused on individual information, such as demographics, water use habits, and medical history. An informed consent was signed by all volunteers prior to completing the questionnaire. The questionnaire was self-administered and was completed by 414 volunteers in the community.

### Study subjects

Of the 414 volunteers, 317 were finally included in our analyses. Questionnaires were excluded from volunteers who were less than 18 years of age (N = 87) because this study was focused on the adult population. In addition, ten individuals with unrealistically high reported daily water consumption (> 5 L day^-1^) were similarly excluded. DM status for the 317 adult volunteers in our sample was based on self-reporting as a response to the question ‘what chronic disease(s) have you been diagnosed with so far by your physician’.

### Arsenic exposure assessment

We utilized data from the questionnaires to compute a cumulative lifetime As exposure (CLAEX) index (mg As) for each participant. The CLAEX index incorporated individual information on drinking-water daily consumption rates and duration of exposure to As in this small community, assuming a lifetime exposure scenario. Exposure duration was set equal to the number of years an individual spent in the As-affected area, knowing that the As-contaminated tap water was the main or exclusive source of drinking-water for > 98% of residents participating in our study. Cypriot population data were obtained from the 2001 CENSUS report of the Statistical Service, Ministry of Finance, Cyprus. The CLAEX index for each individual participant was calculated as (estimated median As concentration in contaminated water, μg L^-1^) × (daily amount of contaminated water consumed from that well, L day^-1^) × (duration of exposure to contaminated water, days), assuming all years living in the As-affected area and consuming water from the well had same consumption rates and same median 40 μg As L^-1^ and with the rest of the years (either outside Mammari or not using the well) assumed to be exposed to a median of 1 μg As L^-1^. This index has been similarly used in Bangladesh [18] and showed no significant (p > 0.05) difference in the prediction of mortality rates between this cumulative As index and that based on a daily As dose as calculated from drinking-water As concentration data. Water consumption rates were individually calculated based on responses about how many glasses of water were daily consumed (based on the notion that on average one glass water equals to ~250 mL water).

### Statistical analysis

CLAEX indices were right-skewed, and hence were logarithmically transformed for the analyses. Arsenic exposure was presented as median with the corresponding interquartile range (IQR) and in order to compare As exposure between different groups we used t-tests and analysis of variance on the log-transformed values. The ratio of geometric means for the cumulative lifetime As exposure index values comparing participants with type II diabetes versus participants without were computed using linear regression models (both unadjusted and adjusted for age, sex, education level, and smoking status) on the log-transformed values of As exposure. This was done for the overall sample, but also for subgroups defined by: age, sex, age, education level, smoking, water use for cooking, and consumption of fish. Logistic regression models were used to model the probability of self-reported diabetes and calculate odds ratios in univariate and multivariate models for several covariates of interest, as well as 95% Wald confidence intervals. Arsenic exposure was included in the logistic regression models as a continuous variable but also in terms of tertiles and quintiles of As exposure. All statistical analyses were performed using SAS 9.1 (SAS Institute, Inc.) and all statistical tests reported were two-sided. Statistical significance was set at p-value <0.05.

## Results

The median (interquartile, IQR) value of CLAEX for all participants in this study was 613 (237–1305) mg As (Table 1). Median male CLAEX indices were similar to those of females (Table 1). Significantly different (p < 0.001) CLAEX indices were observed by age (18–40, 40–60, and >60 years old) with the oldest group having the highest index and the median indices being: 336, 716 and 1483 mg As for each respective age group. Similarly, participants with higher education had significantly (p < 0.001) lower CLAEX indices than those finishing only elementary or high school (Table 1). Approximately 32% of the sampled population was currently smoking, but there was no significant difference (p > 0.05) in CLAEX indices among smokers and non-smokers. A slightly lower % (25%) of current adult (>20 years old) smokers for the whole Cypriot population was recently reported by the Statistical Service of Cyprus [19]. Those participants using the groundwater from the As-contaminated area for cooking (93%) had significantly higher CLAEX indices than those who did not use it (p = 0.03) (Table 1). Up to 87% of our sample in the As-affected region was consuming potable water up to 2.5 L capita^-1^ day^-1^, while the rest 13% consumed water between 2.5 and 4 L capita^-1^ day^-1^ with a median value of 2.0 L capita^-1^ day^-1^ (data not shown). Up to 92% of our sample was exposed to As in potable water for a period up to 60 years, with a median exposure duration value of 16 years (data not shown).

**Table 1:** Median values and their interquartile range values of the cumulative lifetime arsenic exposure indices (CLAEX) calculated for the As-affected study area of Cyprus

	**N** (%)	**Median CLAEX (IQR) (mg As)**	**p-value**
**Total # Participants**	317	613.07 (236.9, 1304.9)	
**Sex**			0.59
Female	167 (53)	634.62 (231.2, 1337.4)	
Male	150 (47)	604.67 (259.1, 1271.1)	
**Age Group**			<.0001
18-40	121 (38)	335.85 (136.6, 635.5)	
40-60	123 (39)	715.89 (259.1, 1314.9)	
60 +	73 (23)	1482.92 (847.0, 1957.7)	
**Education**			<.0001
Elementary	85 (27)	1337.36 (697.6, 1838.1)	
High School	139 (44)	568.88 (275.7, 1183.4)	
Undergraduate	71 (22)	335.85 (138.7, 803.5)	
Post-graduate	22 (7)	349.82 (96.4, 550.6)	
**Smoking**			0.99
No	217 (68)	635.54 (250.9, 1303.9)	
Yes	100 (32)	593.99 (196.0, 1405.3)	
**Use of Water for Cooking**			0.03
No	20 (7)	241.98 (85.3, 1019.0)	
Yes	296 (93)	646.13 (253.9, 1321.2)	
**Diabetes**			0.02
No	296 (93)	573.31 (222.7, 1277.0)	
Yes	21 (7)	998.59 (543.3, 1838.1)	
**Fish Consumption**			0.64
No	79 (25)	731.23 (159.4, 1285.7)	
1-3/week	224 (71)	615.45 (251.8, 1322.6)	
More than 1–3/week	14 (4)	489.30 (221.3, 1227.2)	

Self-reported DM prevalence in our sample from this As-affected region of Cyprus was 6.6%, which is similar to the officially reported DM prevalence for Cyprus of 6.5% by Loizou et al. [17]. Significantly higher (p = 0.02) CLAEX index values were observed for the diabetics (999 mg As) versus the non-diabetics (573 mg As) (Table 1). Reported fish consumption was not associated with CLAEX values as calculated from drinking water in this study. A multi source human As exposure assessment from both seafood and fish, rice and drinking-water in Cyprus suggested that seafood and fish had little influence to the overall lifetime average daily As dose [20], corroborating the fish and seafood consumption results from this study.

Multivariate models adjusted for age, sex, and smoking status showed that participants with DM (though not statistically significant) had 22% higher CLAEX index values (95% CI 0.74, 1.99) than those participants without (Table 2). Subgroup analyses on the adjusted (age, sex and smoking status) ratio of geometric means showed that the association between As exposure and DM cases was lower in those participants with lower educational level, who were males, older, smokers, not using water to cook, and consumed fish between 1–3 times per week (Table 2).

**Table 2:** Ratios of geometric means for the cumulative lifetime arsenic exposure index values comparing participants with type II diabetes versus participants without

	**Unadjusted Ratio of Geometric Means (95% CI)**	Adjusted* Ratio of Geometric Means (95% CI)
**Overall**	1.93 (1.11, 3.39)	1.22 (0.74, 1.99)
**Sex**		
Female	1.99 (0.77, 5.16)	1.57 (0.70, 3.46)
Male	1.88 (0.96, 3.67)	1.11 (0.59, 2.08)
**Age group**		
18-40 years	1.65 (0.28, 9.58)	1.48 (0.25, 8.58)
40-60 years	1.20 (0.51, 2.80)	1.19 (0.50, 2.77)
>60 years	1.13 (0.70, 1.79)	1.26 (0.79, 2.01)
**Education**		
Elementary	1.16 (0.53, 2.53)	1.35 (0.73, 2.48)
High School	1.84 (0.83, 4.14)	1.08 (0.47, 2.51)
Undergraduate	2.36 (0.55, 10.28)	1.51 (0.37, 6.11)
Post-graduate	4.81 (0.25, 93.69)	1.95 (0.11, 34.81)
**Smoking status**		
No	1.63 (0.73, 3.63)	1.25 (0.62, 2.53)
Yes	2.34 (1.05, 5.21)	1.20 (0.58, 2.44)
**Using water to cook**		
No	2.27 (0.25, 20.70)	0.73 (0.08, 6.36)
Yes	2.05 (1.14, 3.71)	1.36 (0.80, 2.29)
**Consumption of Fish**		
No	2.14 (0.62, 7.32)	1.43 (0.45, 4.62)
1-3/week	1.88 (1.01, 3.53)	1.19 (0.69, 2.05)
More than 1-3/week	NA**	

*Adjusted for age, sex, education and smoking status.

** Inadequate available subgroup sample size to run the model.

Age, smoking, and As exposure (log-transformed CLAEX) were significant predictors of increased odds of DM in univariate models (Table 3). Arsenic exposure was associated with an almost 80% increase in the odds of DM (OR = 1.78; 95% CI: 1.08, 2.93). Sex and education were not significant predictors of increased odds of DM. The unadjusted OR for DM comparing participants in the 80^th^ vs. the 20^th^ percentiles of total As exposure (CLAEX index) was 5.00 (95% CI: 1.03, 24.17).

**Table 3:** Univariate and multivariate models predicting the odds ratios of developing diabetes mellitus among the participants of the study area in Cyprus

**Univariate Models**	**OR (95% CI)**	**p-value**
**Sex – Males vs. Females**	1.876 (0.75, 4.66)	0.18
**Age (year)**	1.047 (1.02, 1.08)	0.001
**Smoke – Yes vs. No**	2.563 (1.05, 6.25)	0.04
**Log arsenic exposure (mg)**	1.777 (1.08, 2.92)	0.02
**Education**		
High School vs. Elementary	0.672 (0.25, 1.81)	0.43
Undergraduate vs. Elementary	0.431 (0.11, 1.69)	0.23
Postgraduate vs. Elementary	0.481 (0.06, 4.07)	0.50
**Tertiles of arsenic exposure**		
Second vs. First	4.781 (1.01, 22.69)	0.05
Third vs. First	5.368 (1.15, 25.13)	0.03
**Quintiles of arsenic exposure**		
Second vs. First	0.968 (0.13, 7.09)	0.97
Third vs. First	2.632 (0.49, 14.11)	0.26
Fourth vs. First	1.500 (0.24, 9.30)	0.66
Fifth vs. First	5.000 (1.03, 24.17)	0.04

**Multivariate Models**	**OR (95% CI)**	**p-value**
**Model 1**		
Age (year)	1.036 (1.00, 1.07)	0.04
Males vs. Females	1.280 (0.43, 3.84)	0.66
Smoke – Yes vs. No	2.034 (0.69, 5.99)	0.20
Log arsenic exposure (mg)	1.287 (0.74, 2.25)	0.38
**Model 2**		
Age (year)	1.041 (1.00, 1.08)	0.02
Males vs. Females	1.268 (0.42, 3.81)	0.67
Smoke – Yes vs. No	2.000 (0.68, 5.91)	0.21
Tertiles of arsenic exposure		
Second vs. First	3.493 (0.70, 17.33)	0.13
Third vs. First	2.287 (0.41, 12.66)	0.34
**Model 3**		
Age (year)	1.038 (1.00, 1.08)	0.03
Males vs. Females	1.425 (0.47, 4.29)	0.53
Smoke – Yes vs. No	1.867 (0.63, 5.51)	0.26
Quintiles of arsenic exposure		
Second vs. First	0.717 (0.09, 5.56)	0.75
Third vs. First	1.791 (0.31, 10.26)	0.51
Fourth vs. First	0.774 (0.109, 5.516)	0.80
Fifth vs. First	1.857 (0.298, 11.592)	0.51

In multivariate models adjusted for age, sex, and smoking, As exposure effects on DM categorized in quintiles was no longer statistically significant (Table 3).

## Discussion

This is the first study evaluating the relationship between human As exposures (via the consumption of contaminated groundwater) and the prevalence of DM in an As-impacted region of Cyprus. We conducted this study in a specific rural area of Nicosia, Cyprus where As contamination of groundwater was discovered in June 2009. In the absence of As human biological markers of exposure, we used a CLAEX As exposure index, which has also been used in large prospective cohort studies in As endemic areas of the world (Bangladesh) [18]. We found that increasing cumulative lifetime exposure to As, as measured by the CLAEX index, was associated with increased odds of DM in unadjusted models. However, given the relatively small sample size of this study, the confidence intervals were wide and, after adjustment for several important covariates such as age, were no longer statistically significant.

Nonetheless, this study provides important information for human health risk assessment in the region. Epidemiologic data on diabetes in Cyprus are scarce and prevalence estimates for adults > 20 years old range from 5.1% to 10.3%. The EU-wide average prevalence of DM is around 7.5% [21] while the U.S. prevalence of DM is 7.7% according to data from the National Health and Nutrition Examination Study (NHANES) [15]. Sex-specific population age distribution patterns were quite similar between the study area and that of the whole Cyprus (data not shown). The mean DM prevalence of the diagnosed cases in the study area (6.6%) was similar to that for Cyprus as a whole (6.5%) [17], suggesting that our study population is not different in this respect than the Cypriot population in general.

Low-level As exposure studies focusing on the risk of chronic diseases, such as DM, are limited worldwide and, thus, a causal association between low-level As exposure and the prevalence of DM has yet to be confirmed. Recent mechanistic data imply that low-level As exposures provoke a cellular adaptive oxidative stress response involved in glucose-stimulating insulin secretion, and eventually disturbed β-cell function [22]. However, it is also true that having a chronic disease, such as DM, may alter As metabolism and thus lead to higher exposures among diseased individuals.

According to recent data obtained from regulatory agencies in Cyprus, As concentrations in groundwater in this study were typically < 50 μg L^-1^. The lack of adequate As exposure assessment estimates due to absence of a systematic and network-wide well-water As concentrations led us to use the median value of 40 μg L^-1^, which may overestimate human exposures in the affected region. However, our post sensitivity analysis using a median value of 20 μg L^-1^ did not significantly (p > 0.05) change the univariate and multivariate-based OR of DM and trends observed in this study (data not shown). Thus, this study provides adequate information on the association between low-levels As exposure and risk of developing DM. The collection of individual data on daily drinking water consumption as well as the exposure duration and adjustment of several DM risk factors strengthened the results of this study.

### Study limitations

Despite these important aspects of this study, there were several limitations. In particular, the small sample size (317 participants) limited the statistical power to observe an effect of low As exposure on DM. Several important risk factors for DM, such as family history of DM and BMI, were not available, thus limiting our ability to address relative contributions of these factors to the observed OR of DM and to better estimate the relative contribution of As exposure to DM after adjusting for these factors. Epidemiologic studies on the association of low-level (< 100 μg As L^-1^) As exposure and the risk of developing lung and bladder cancer were limited in their ability to detect the predicted estimates of excess risk because of sample size and less than lifetime exposure [23]. Additional studies with larger sample sizes are needed to lower the magnitude of uncertainty in the conclusions.

Another limitation of this study was the absence of biomarker As data, such as toenails, elucidating historic As exposure patterns. Toenail As data provides historic evidence of As exposure via both drinking water and food diet exposure pathways [24-27]. However, we used a cumulative exposure index as a surrogate of lifetime As exposure to drinking water that has previously been used in large epidemiologic studies. Recent studies have observed a highly significant (p < 0.001) positive correlation between toenail As concentrations and As concentrations in drinking water [26,27]. From a study conducted by the Cypriot Ministry of Health that we participated in, preliminary data were collected for a small case–control group of people (N = 65 for cases and similar number for the control group) comparing those in the As-affected area with those in the unaffected area. Toenail As concentrations were significantly higher (p < 0.001) in the As-affected area compared to those in an As-unaffected area of Cyprus (unpublished).

Dietary As intake, via the consumption of seafood, may also contribute to the overall As daily dose [[27]]. In Bangladesh, at drinking water As concentrations > 50 μg As L^-1^, drinking water was the dominant route of exposure to humans, whereas at As concentrations < 50 μg As L^-1^, the dominant route of exposure was dietary [26,27]. Food items that are cooked in As-contaminated water may absorb As at the cooking temperature and, thus, enter the food chain [26,27]. Epidemiologic studies in the U.S. based on NHANES data showed that controlling for nontoxic forms of As (arsenobetaine in seafood) aided in better illustrating the inorganic As effect on human health [15]. The U.S. EPA defines a reference dose level for As of 0.3 μg As kg^-1^ day^-1^ for a 70 kg adult, but, interestingly, increased risk for DM was shown for doses less than this reference dose [15]. Mean daily dietary inorganic As intake (DII) for Cypriots (from fish/seafood, rice and water) with average 5 μg As L^-1^ drinking-water was 0.2 μg kg^-1^ capita^-1^ day^-1^[20]. Seafood and fish consumption was not a significant predictor of DM, paralleling results from a recent probabilistic exposure assessment conducted for the Cypriot population [20]. Results from that study showed that fish, seafood and rice consumption in Cyprus contributed <25% of DII, while drinking water contributed >75% of DII [20], despite the documented high consumption rates of fish/seafood and rice in Cyprus. It, thus, follows that focusing on drinking-water, instead of dietary items, is adequate in estimating overall inorganic As exposure. The DII global estimates typically use a median value of 2.0 L capita^-1^ day^-1^ water consumption rate estimates, which is similar to that estimated in our As-affected study area.

In summary, well-designed epidemiologic studies for low-level As exposure scenarios, such as that encountered in Cyprus, are lacking. Small communities (< 2000 residents), such as the As-affected area of Cyprus, that typically rely on groundwater to satisfy their potable needs are widespread throughout the globe. These communities may lack centralized drinking water treatment facilities that could provide them with contaminant-free drinking water. This study highlights the importance of conducting detailed As exposure assessment and risk calculations in such communities. The spatial and temporal variability of groundwater As contamination in Cyprus is currently unknown and regulatory agencies of Cyprus are interested in further looking into it. Future research is envisioned in accurately measuring the uncertainty of As external and internal exposure measurements for different areas of Cyprus, while attempting to quantify the relationship between As exposures and DM by recruiting a larger volunteer size. The completion of a human health risk assessment based on the annual total social costs for alternative As intervention measures is expected in the near future.

## Conclusions

This preliminary assessment of environmental risk factors, such as As, on the etiology of DM will generate guidelines and recommendations for similarly As-affected rural areas in Cyprus and in the Eastern Mediterranean region. Arsenic-impacted communities are in need of exposure-, and epidemiologic-based information to identify proper remediation plans and ways to minimize As-related morbidity. Diabetes prevention programs have mostly focused on medication and lifestyle modification, but the role of environmental exposures must also be considered.

## Competing interests

The authors declared that they have no competing interests.

## Authors’ contributions

KCM coordinated study execution, questionnaire collection and data digitization along with drafting the manuscript. CAC performed all statistical analyses along with MP. ASE facilitated interpretation of results and contributed in writing the manuscript. All authors read and approved the final manuscript.

## Pre-publication history

The pre-publication history for this paper can be accessed here:

http://www.biomedcentral.com/1471-2458/12/334/prepub
